# Medicinal dietary plants of the Yi in Mile, Yunnan, China

**DOI:** 10.1186/s13002-020-00400-5

**Published:** 2020-08-28

**Authors:** Jingxian Sun, Yong Xiong, Yanhong Li, Qingsong Yang, Yijian Chen, Mengyuan Jiang, Yukui Li, Hongrui Li, Zizhen Bi, Xiangzhong Huang, Shugang Lu

**Affiliations:** 1 Institute of Ecology and Geobotany, School of Ecology and Environmental Science, Yunnan University, Kunming, 650091 P. R. China; 2 Key Laboratory of Chemistry in Ethnic Medicinal Resources, State Ethnic Affairs Commission & Ministry of Education, School of Ethnic Medicine, Yunnan Minzu University, Kunming, 650500 P. R. China; 3 College of Life and Environmental Sciences, Minzu University of China, Beijing, 100081 P. R. China

**Keywords:** Ethnobotanical surveys, Mile City, Yi people, Traditional medicine

## Abstract

**Background:**

The Yi is the largest ethnic group in Yunnan Province (China), with a population of five million. The Yi people tend to live in mountainous areas, and their culture includes a unique dietary system for treating and protecting people against illnesses. Medicinal plants occupy an essential place in the Yi diet because they play a key role in health and the prevention and treatment of diseases. However, few studies have addressed these medicinal dietary plants and their importance in the Yi’s daily lives. The aim of this study was to (1) investigate the medicinal dietary plants used by the Yi in Mile City, (2) document the traditional knowledge held about these plants, (3) identify species with important cultural significance to the Yi in Mile City, and (4) analyze the special preparation methods and consumption habits of these plants.

**Methods:**

Field investigations were performed in six villages in Mile City, Honghe Hani and Yi Autonomous Prefecture, Yunnan, from July 2017 to May 2018. Information was collected using direct observation, semi-structured interviews, key informant interviews, individual discussions, and focus group discussions. The use value (UV) and frequency of utilization index (FUI) of these plants were analyzed. Plant samples and voucher specimens were collected for taxonomic identification.

**Results:**

This study documented 124 species belonging to 62 families and 102 genera. These plants included angiosperms (117 spp.), gymnosperms (3), pteridophytes (2), lichen (1), and fungus (1). The 20 species with the highest UV were noted as being particularly important to the Yi people’s daily life in Mile City. The primary medicinal preparation method for plants recorded in the study was decoction. The most commonly used plant parts were fruits and roots. The most frequently used edible parts were fruits, and the most frequently used medicinal parts were roots. The medicinal parts were used to treat diseases such as rheumatism, edemas, kidney deficiency, spleen deficiency, gastritis, parasites, and so on.

**Conclusion:**

A wide variety of medicinal dietary plants are used by the Yi people in Mile City. Those plants, which have both rich nutritional and medicinal value, occupy an essential part of the Yi dietary and medicine culture. Ethnobotanical surveys of medicinal dietary plants provide a theoretical reference for the conservation and sustainable use of the plant resources and could contribute to the protection of the Yi food culture and traditional medicine in Mile City. In addition, this information provides a sound basis for developing and using Yi ethnic medicine and health products.

## Background

“Medicinal dietary plants” refer to plants that can be eaten and also used as medicine to prevent and cure diseases, and include health plants [1]. There are many overlaps between medicine food, and dietary products can simultaneously be food medicine [2]. In fact, many plants in local food cultures have therapeutic value. The concept of “medicinal dietary” use is based on ancient lore about food medicine discovery in ancient times, which reflected the edibility and medicinal function of certain plants. It first appeared in the form of “food and nutrition,” “food therapy,” and “tonic food,” emphasizing the role of both medicinal and edible resources in health care, prevention, and the auxiliary treatment of diseases [3]. In many traditions in India, the species used as medicines are also used as food and vice-versa; in many cases, ethnobiologists have documented this unclear delineation between food medicine [4, 5]. Throughout the world, people are emphasizing health care and health preservation, and they are advocating natural cures. In western countries, some people propose the use of “kitchen instead of pharmacy” and “food instead of medicine” [6]. In recent years, with the general improvement in people’s living standards, knowledge about dietary hygiene and nutrition has become more widespread. People in China pay more and more attention to their health. The health care concept of “medicine food is of the same origin” and “medicine food is of the same function” has gradually gained popularity, and it has even affected countries and regions such as Japan and Southeast Asia. Yu Rensheng, a famous traditional Chinese medicine health care company, is one typical example [3, 7].

*Shen Nong*’*s Herbal Classic*, the first mainstream herbology monograph in China, recorded many medicinal dietary plants. Since 1985, more than 10 food therapy books have been published per year in China [8]. However, the study of medicinal dietary plants used by indigenous communities in China has largely been neglected. The Yi, one of the most ancient ethnic groups in Southwest China [9], is the sixth largest ethnic minority [10], and their population is primarily distributed throughout Yunnan, Guizhou, Sichuan, and Guangxi provinces in Southwest China, with a population of approximately 8.71 million. Approximately 61% of the Yi people live in Yunnan Province [11]. Two autonomous prefectures, Chuxiong and Honghe, and another 15 autonomous counties, including Nanjian, Luquan, and Shilin, are the primary locations for the Yi people in Yunnan Province. The rise in Yi medicine in the southwest can be traced to the Eastern Han Dynasty, 1800 years ago [9]. In the long struggle against disease and harsh environments, a specific system of food medicine was developed by the Yi [12]. For example, many medicinal plants are used not only for essential components of the daily diet but also play an important role in health care and disease prevention under conditions of limited medical resources [13–18]. *Xian Yao Ching* of Yi ethnicity wrote during the Qing Dynasty (AC 1636) that all vegetables and plants could be used as medicinal materials. Plants such as *Amomum tsao*-*ko*, *Zingiber officinale*, and *Piper nigrum* were reported to show therapeutic efficacy. Furthermore, all plants, animals, livestock materials, and grains used as medicines could be administered in combination with one another to improve their curative effects. Previously, some records about the medicinal plants of the Yi were documented in publications such as *Yi Herbal Medicines* [19], *Theory and Application of Yi Medicine* [20], and *Yi Medicines of Chinese* [21]*.*

Although ethnobotanical surveys on medicinal dietary plants in the Lijiang, Xishuangbanna, Jinfoshan, and Taibai Mountain areas in China, and the Vulture area in southern Italy, have been published [22–24], there has been no equivalent study on the food culture of the Yi people in the Honghe Hani and Yi Autonomous Prefectures. Therefore, this study undertook ethnobotanical surveys on the medicinal dietary plants used by the Yi people in Mile City. By investigating the retention of traditional knowledge regarding medicinal dietary plants as related through the experiences of the Yi people in Mile City, Yunnan, we aim to (1) investigate the medicinal dietary plants used by the Yi in Mile City, (2) document the traditional knowledge held about these plants, (3) identify species with important cultural significance to the Yi in Mile City, and (4) analyze their special preparation methods and consumption habits of these plants. Our findings may provide references for biodiversity protection, rational development, and the sustainable use of Yi medicinal dietary plant resources and traditional medicinal dietary knowledge.

## Methods

### Study area

**Fig. 1:**
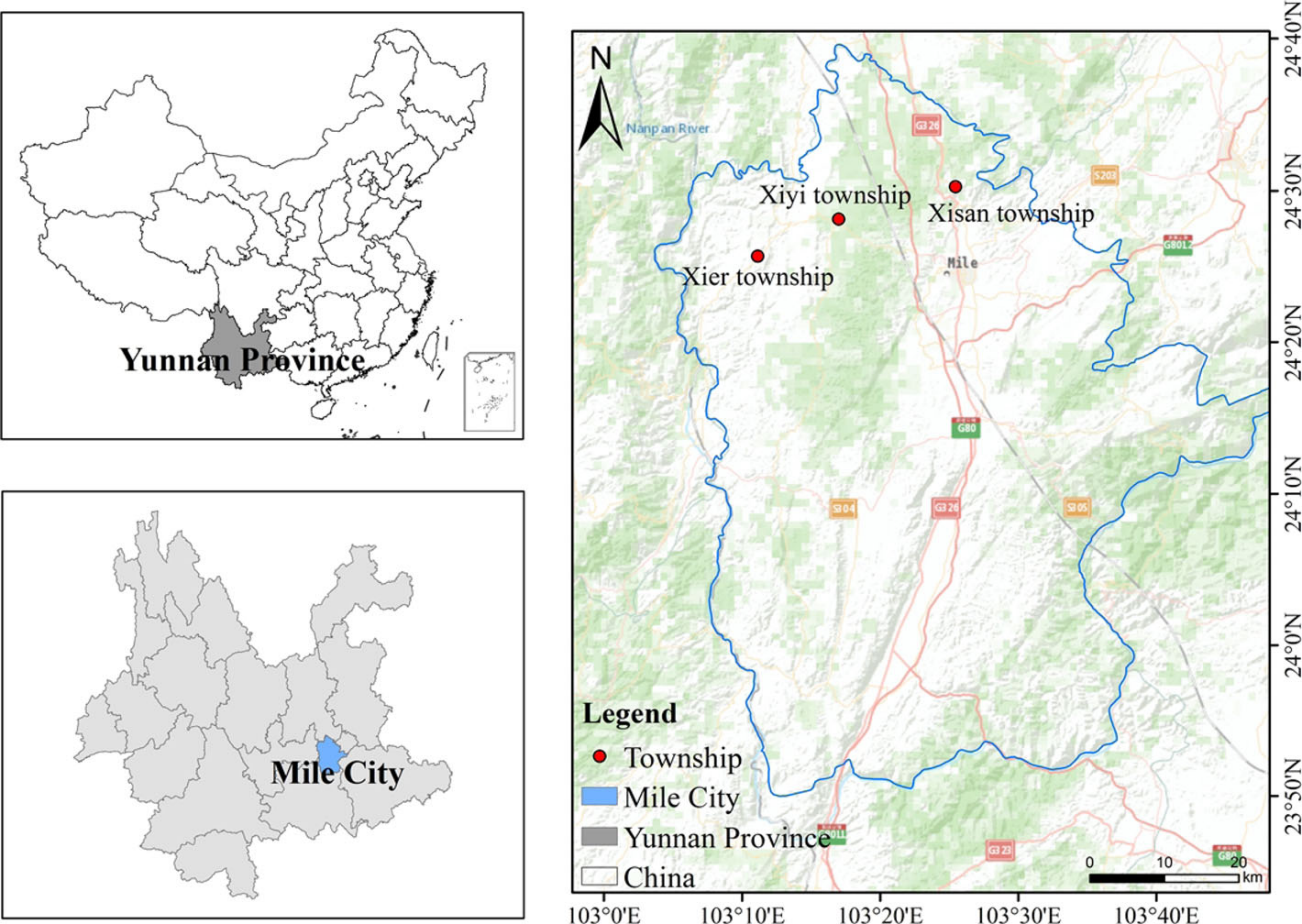
Location of the area covered in the investigation into medicinal dietary plants used by Yi in Mile City, Yunnan, China

Honghe Hani and Yi Autonomous Prefecture is located in Southeastern Yunnan Province, China, with Wenshan to the east, Kunming to the north, Yuxi to the west, and Vietnam to the south [25]. Mile (103° 04′–103° 49′ E and 23° 50′–24° 39′ N) is a county-level city situated in the northern part of the Autonomous Prefecture and is composed of 12 townships (Fig. 1). Mile City is known as the north gate of Honghe; it is approximately 78 km from east to west, 50 km in the south-north direction, and covers a total area of 4004 km^2^. Mile City is in a subtropical monsoon climatic zone with high elevations in the north and low elevations in the south and has a population of 527,767, including approximately 200,000 Yi people. The highest area in Mile City is Jinding Mountain, with an altitude of approximately 2315 m, which is located east of Xinshao, and the lowest point is the egress of the Nanpan River, with an altitude of approximately 862 m. The climate of Mile City is primarily dominated by plateau monsoons, with a mean annual temperature of 18.8 °C and a mean annual rainfall of 835.4 mm [25].

### Data collection

**Fig. 2:**
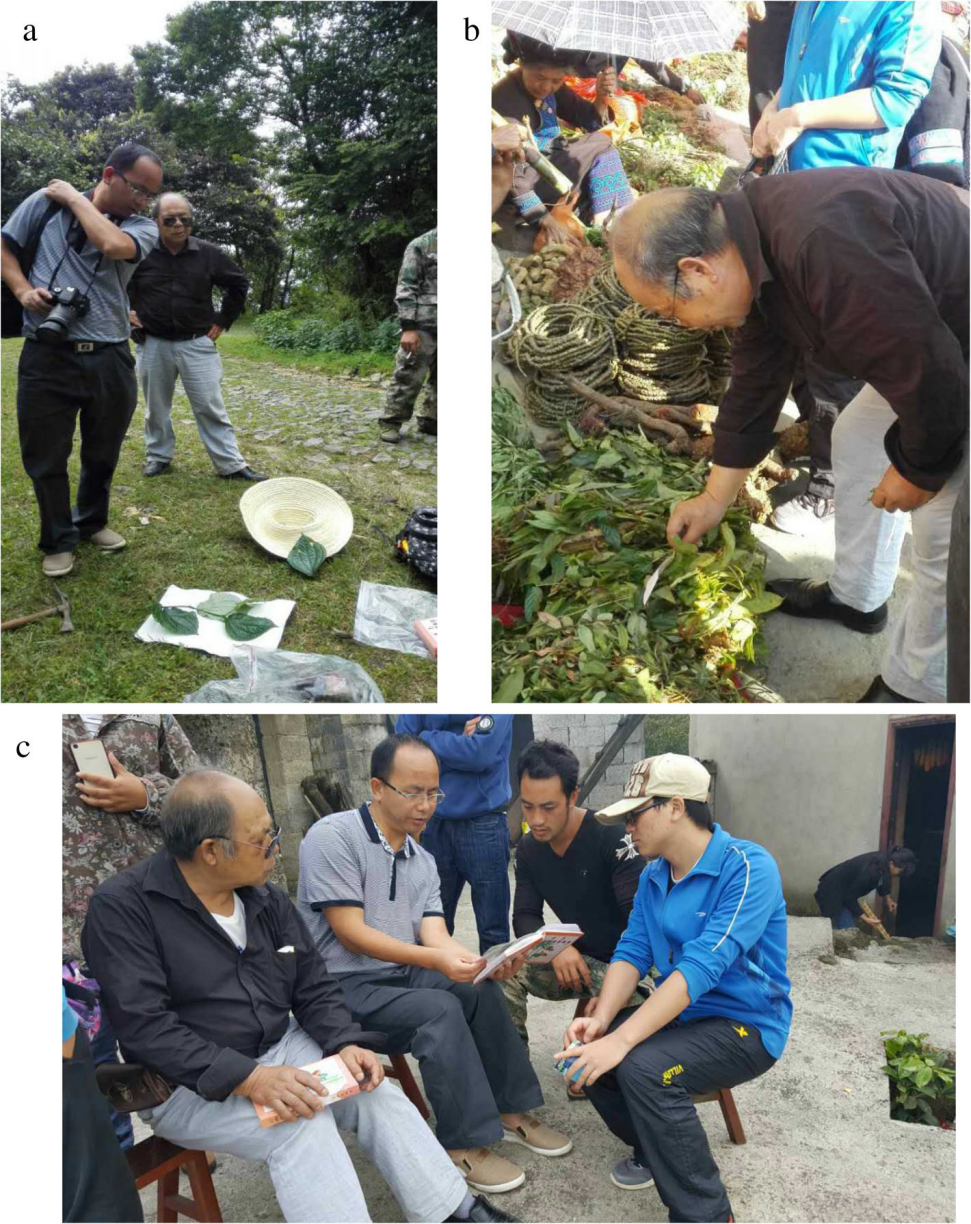
Medicinal dietary plants collecting in local areas **a** One of the authors collected medicinal dietary plants in a village **b** Local herbal medicinal markets **c** Focus group discussions

Field studies were performed during three visits from July 2017 to May 2018. This study was performed in accordance with the guidelines from the International Society of Ethnobiology Code of Ethics (http: //www.ethnobiology.net) [26] and the American Anthropological Association Code of Ethics (https://www.americananthro.org) [27]. Thirty-six key informants, who had considerable knowledge and experience regarding the use of medicinal dietary plants, were selected for the interviews, including eight healers. Most of them had acquired medical treatment skills and knowledge from their parents. The investigated localities covered six villages in three townships (Xiyi, Xier, and Xisan). Ethnobotanical data were collected through direct observation, semistructured interviews, key informant interviews, individual discussions, and focus group discussions (Fig. 2) [28–30]. In the present study, the Yi names, local names, Latin names, edible parts, medicinal parts, preparation methods, and efficacy of the plants were recorded. Some information on Yi ethnic medicine and food culture was also recorded. The research focused exclusively on medicinal dietary plant use and knowledge. All the interviews were conducted with each interviewee’s consent. Generally, the participants were required to answer the following questions:

1. Which medicinal dietary plants do you use?
2. How do you consume these plants?
3. Do you use one part of the plant for food and another part for medicine?
4. Do you have any special preparation methods?
5. When do you collect medicinal dietary plants?
6. Where do medicinal plants grow around your community?
7. How do the Yi people conserve medicinal dietary technologies, associated cultural practices, and traditional knowledge?

### Data analysis

Most of the Yi people in Mile City, especially official workers, students, and traders, can speak basic Mandarin. Therefore, interviews were conducted in Mandarin rather than through interpreters. When describing the plants, the participants used vernacular names. Voucher specimens of the medicinal dietary plants have been collected by the first author during survey, and voucher specimens were examined and identified by Dr. Qingsong Yang and Dr. Zizhen Bi in laboratory. Latin names were recorded by referring to *The Plant List* (http://www.theplantlist.org/) [31]. Specimens were deposited in a herbarium of Key Laboratory of Chemistry in Ethnic Medicinal Resources, State Ethnic Affairs Commission & Ministry of Education, School of Ethnic Medicine, Yunnan Minzu University, Kunming, China.

The data collected in the Mile area were collated into an inventory listing all the medicinal dietary plants and related information. The use value (UV) of each medicinal dietary plant was calculated to evaluate the relative importance of each plant based on the number of times it was cited and the number of informants [32, 33]. The formula for the UV is

$$ \mathrm{UV}=\left(\sum Ui\right)/N $$

where *Ui* is the number of times cited by each informant for a certain medicinal dietary plant and *N* is the total number of informants [34]. The frequency of utilization index (FUI) for medicinal dietary plant species was graded according to the frequency of consumption by local people. The FUI can also reflect the degree of closeness between the medicinal dietary plant species and the local community. The FUI scores range from 0 to 5 and vary according to the consumption frequency (Table 1) [34].

**Table 1:** The FUI value and corresponding category

Consumption frequency	FUI
More than once a week	5
Once a week	4
Once a month	3
More than once a year, less than once a month	2
Once a year	1
No consumption in last 30 years	0.5

## Results

### Documented medicinal dietary species

Our survey showed that medicinal dietary plants were widely used by the Yi people in Mile City. In total, 124 species including angiosperms (117 spp.), gymnosperms (3), pteridophytes (2), lichen (1), and fungus (1) were documented (Tables 2 and 3). Detailed information about the plants was displayed in Table 2 (plants mentioned only by one informant were not documented in Table 2). The average number of species mentioned per informant was approximately nine. Plants belonging to 62 families and 102 genera were classified as different life forms, including herbs (47.6%), trees (27.4%), vines (10.5%), and shrubs (12.9%). The majority of the plants belonged to the Rosaceae (18 species), Lamiaceae (6), Leguminosae (6), Compositae (5), Araliaceae (5), Amaryllidaceae (4), and Cucurbitaceae (4) families. The genera with the highest number of species were *Allium* (4 species), and *Prunus* (4), followed by *Diospyros* (3), *Fragaria* (3), *Elsholtzia* (3), and *Rubus* (3).

**Table 2:** Medicinal dietary plants used by the Yi people in Mile City, Yunnan Province, China

Scientific name, Family, Collector, Voucher number	Vernacular name	Yi name	Life form	Medicinal parts	Preparing methods	Medicinal uses	Edible parts	Cooking methods	FUI	UV
*Amaranthus tricolor* L., Amaranthaceae, XZH-20171037	Zilingxian	Nuosongnibaizai	H	WP	Decoction	Clearing the liver and improving vision	WP	Stir fried or boiled	2.1	0.42
*Chenopodium album* L., Amaranthaceae, XZH-20171017	Huitiaocai	Nisewu	H	WP	Decoction	Clearing the liver and improving vision	L and ST	Stir fried or boiled	2.8	0.54
*Allium macrostemon* Bunge, Amaryllidaceae, XZH-201710100	Xiaopusuan	Songpu	H	WP	Decoction	Parasites; reducing phlegm	WP	Stir fried, boiled, or pickled	2.2	0.42
*Allium sativum* L., Amaryllidaceae, XZH-201710469	Dasuan	Shutongzai	H	WP	Decoction	Rhinitis; parasites	BU	Flavoring	4.7	0.90
*Allium scorodoprasum* L., Amaryllidaceae, XZH-201710248	Xiaosuan	Shubuazeizai	H	WP	Decoction	Parasites; fever	BU	Flavoring	2.1	0.47
*Allium wallichii* Kunth, Amaryllidaceae, XZH-201710120	Shanjiucai	Caiguageizai	H	WP	Stir-fried with eggs	Lactation stimulant	WP	Boiled or pickled	2.3	0.50
*Dobinea delavayi* (Baill.) Baill., Anacardiaceae, XZH-20171059	Yangjiaotianma	Ciwochengnongcizai	H	RH	Stewed with chicken	Detumescence; apocenosis; dizziness	RH	Stewed with chicken	1.9	0.45
*Foeniculum vulgare* Mill., Apiaceae, XZH-20171090	Xiaohuixiang	Mengsongmabizai	H	RO	Decoction	Traumatic injury, abdominal distension, and stomachaches	TL	Stir fried or boiled	3.1	0.62
*Ligusticum striatum* DC., Apiaceae, XZH-201710213	Chuanxiong	Wujiegeimizai	H	RH	Stir-fried with eggs	Headaches	RH	Boiled	2.3	0.46
*Oenanthe javanica* (Blume) DC., Apiaceae, XZH-201710513	Shuiqincai	Yuxiangqin	H	WP	Decoction	Hypertension	L and ST	Cold dish or boiled	4.2	0.83
*Cynanchum otophyllum* C. K. Schneid., Apocynaceae, XZH-201710503	Qingyangshen	Cinazai	V	RO	Stewed with chicken	Tranquillizer; epilepsy	FR	Eaten fresh	2.0	0.50
*Marsdenia tenacissima* (Roxb.) Moon, Apocynaceae, XZH-20171099	Tongguangsan	Citongmiluozai	V	RO and ST	Stir-fried with eggs	Coughs; lactogenesis; diuresis	F and TS	Stir fried	1.4	0.37
*Amorphophallus konjac* K. Koch, Araceae, XZH-20171093	Moyu	Mayumezai	H	T	Decoction	Pneumonia; edemas; phlegm; bronchitis; coughs	T	Konjak tofu or boiled	4.9	0.93
*Colocasia esculenta* (L.) Schott, Araceae, XZH-20171077	Qingyu	Abupa	H	T	Decoction	Tonifying the kidney; tonifying the spleen	T, F, and L	Boiled, stir fried, or pickled	2.3	0.39
*Aralia chinensis* L., Araliaceae, XZH-20171022	Congmu	Heiahuozai	T	BA and RO	Decoction	Tonifying the liver; rheumatism	TS	Stir fried or boiled	2.8	0.45
*Eleutherococcus senticosus* (Rupr. & Maxim.) Maxim., Araliaceae, XZH-20171001	Ciwujia	Ziguzai	S	ST and RO	Decoction	Tonifying the kidney; rheumatism	TS	Stir fried or boiled	2.3	0.40
*Hydrocotyle javanica* Thunb., Araliaceae, XZH-201710264	Maticao	Maokongwuzai	H	WP	Roots stewed with pork tripe	Hepatitis A	L	Cold dish	2.1	0.41
*Metapanax delavayi* (Franch.) J. Wen & Frodin, Araliaceae, XZH-20171010	Liangwangcha	Laibuluoyuzai	S	L	Soaked in boiling water	Iaryngopharyngitis	TL	Soaked in boiling water	2.0	0.40
*Panax notoginseng* (Burkill) F.H.Chen, Araliaceae, XZH-20171016	Sanqi	Shenglengzai	H	RO	Stewed with chicken	Nourish blood; promoting blood circulation to remove blood stasis	RO	Stewed with chicken	2.5	0.55
*Trachycarpus fortunei* (Hook.) H. Wendl., Arecaceae, XZH-201710169	Zongshuhua	Situomizai	T	RO, ST, and FR	Decoction	Hypertension; dizziness	F	Stir fried or boiled	2.5	0.48
*Saruma henryi* Oliv., Aristolochiaceae, XZH-201710207	Matixiang	Gongbunizai	H	RH and RO	Stewed with chicken or pig trotter	Tonifying the spleen; stomachaches	RH and RO	Stewed with chicken or pig trotter	1.9	0.45
*Polygonatum odoratum* (Mill.) Druce, Asparagaceae, XZH-201710531	Yuzhu	Medaoweilongzai	H	RH	Stew with eggs or meat	Tonifying the kidney	RH	Stew with eggs or meat	2.2	0.47
*Hemerocallis citrina* Baroni, Asphodelaceae, XZH-201710155	Xuancao	Miluoshanwuzai	H	RO and F	Decoction or Scrambled eggs with flowers	Gastritis	F	Stir fried or boiled	2.7	0.52
*Auricularia auricula* Judae, Auriculariaceae, XZH-20171082	Muer	Rimelong	F	FB	Stir-fried or boiled	Deafness; hypertension	FB	Stir fried or boiled	4.2	0.89
*Capsella bursa-pastoris* (L.) Medik., Brassicaceae, XZH-201710134	Jicai	Mengjiewu	H	WP	Decoction	Nephritis; edemas	L	Stir fried or boiled	2.9	0.57
*Adenophora stricta* Miq., Campanulaceae, XZH-201710455	Shashen	Yanpubiguozai	H	RO	Stewed with chicken	Tonifying the kidney; moistening the lung	RO	Stewed with chicken	1.8	0.42
*Codonopsis cordifolioidea* P. C. Tsoong, Campanulaceae, XZH-201710226	Choushen	Yanmebinengzai	V	RO	Stewed with chicken	Tonifying the kidney; strengthening yang-qi	RO	Stewed with chicken	2.7	0.58
*Cannabis sativa* L., Cannabaceae, XZH-201710461	Huomazi	Zime	H	SE and RO	Made oil	Deobstruent; moistening the lung	SE	Made oil	2.5	0.54
*Lonicera japonica* Thunb., Caprifoliaceae, XZH-20171042	Jinyinhua	Tongshanmilongzai	V	F, L, and ST	Decoction	Fever; coughs	F	Stir fried or boiled	3.1	0.60
*Silene viscidula* Franch., Caryophyllaceae, XZH-201710345	Wacao	Gawuwu	H	RO	Decoction	Acne, coughs, and reducing sputum	L	Stir fried or boiled	1.9	0.40
*Arctium lappa* L., Compositae, XZH-201710417	Niubangzi	Nianbamezai	H	RO	Stewed with chicken	Gastric disorders; tinnitus	RO	Stewed with chicken	2.0	0.58
*Cirsium arvense* (L.) Scop., Compositae, XZH-201710546	Xiaoji	Shanpaicimeazizai	H	RO	Stewed with meat	Tonifying the kidney; detumescence	RO	Stewed with meat	1.7	0.41
*Cirsium japonicum* (Thunb.) Fisch. ex DC., Compositae, XZH-201710523	Dajijiaoshu	Shanpaicimezai	H	RO	Stewed with meat	Tonifying the kidney; detumescence	RO	Stewed with meat	1.8	0.38
*Cornus kousa* subsp. *chinensis* (Osborn) Q.Y.Xiang Cornaceae, XZH-201710216	Jisuzi	Sanzimezai	T	L and RO	Decoction	Ascaricide; acaricide	TS and FR	Shoot stir fried; ripe fruits eaten fresh	1.3	0.43
*Cucurbita moschata* Duchesne, Cucurbitaceae, XZH-20171032	Nangua	Apumezai	H	SE	Decoction	Ascaricide	FR and SE	Fruit boiled; Seeds stir fried	4.9	0.93
*Gynura bicolor* (Roxb. ex Willd.) DC., Compositae, XZH-20171013	Guanyincai	Buwuzai	H	WP	Decoction	Hepatitis; improving vision	TP	Stir fried or boiled	1.2	0.30
*Taraxacum mongolicum* Hand.-Mazz., Compositae, XZH-201710485	Pugongying	Wujieshandongzai	H	WP	Stir fried with eggs	Analgesic; diuresis	WP	Stir fried or boiled	4.5	0.89
*Merremia hungaiensis* (Lingelsh. & Borza) R. C. Fang, Convolvulaceae, XZH-201710377	Shantugua	Agaizai	H	RO	Decoction	Tonifying the spleen; abate jaundice	FR and RO	Boiled or stir fried	2.0	0.49
*Sechium edule* (Jacq.) Sw., Cucurbitaceae, XZH-20171084	Yangsigua	Lianbugua	V	RO	Decoction	Gastric disorders; colitis	TS and FR	Stir fried or boiled	4.5	0.88
*Solena amplexicaulis* (Lam.) Gandhi, Cucurbitaceae, XZH-20171002	Laoshuhuanggua	Mudengmilongzai	H	RO	Decoction	Pneumonia; coughs	FR	Ripe fruits eaten fresh	1.6	0.43
*Trichosanthes Kirilowii* Maxim., Cucurbitaceae, XZH-201710443	Tianhuafen	Mudengazizai	V	RO	Decoction	Relieving asthma; coughs and reducing sputum	FR	Ripe fruits eaten fresh	2.1	0.43
*Pteridium aquilinum* (L.) Kuhn, Dennstaedtiaceae, XZH-201710381	Juecai	Abiwujiezai	H	Rh	Decoction	Angina pectoris	TL	Stir fried or boiled	2.7	0.47
*Dioscorea alata* L., Dioscoreaceae, XZH-201710198	Maoshu	Abunibuzai	V	T	Stewed with meat	Tonifying the spleen; indigestion	T	Boiled or stewed	2.2	0.42
*Dioscorea subcalva* Prain & Burkill, Dioscoreaceae, XZH-201710312	Yeshanyao	Laigeiageizai	V	T	Stewed with meat	Tonifying the spleen; tonifying the kidney	T	Boiled or stewed	1.6	0.34
*Diospyros kaki* L. f., Ebenaceae, XZH-20171044	Shizishu	Sanbaomezai	T	RO	Decoction	Anthelmintic; vomiting	FR	Ripe fruits eaten fresh	2.3	0.55
*Diospyros kaki* var. *silvestris* Makino, Ebenaceae, XZH-201710397	Yeshizi	Laigusanbazai	T	RO	Decoction	Enteritis; cystitis	FR	Ripe fruits eaten fresh	2.4	0.51
*Diospyros lotus* L., Ebenaceae, XZH-20171035	Ruanzao	Sanbaniguozai	T	RO	Decoction	Diuresis; expelling toxins	FR	Ripe fruits eaten fresh	2.2	0.46
*Rhododendron mucronatum* (Blume) G. Don, Ericaceae, XZH-201710237	Baihuadujuan	Caigumiluotongzai	S	RO	Decoction	Cystitis; hepatitis	F	Stir fried or boiled	2.6	0.57
*Castanea mollissima* Blume, Fagaceae, XZH-201710366	Banli	Ganme	T	FR and RO	Decoction	Tonifying the kidney; sialaporia	FR	Stir fried or boiled	2.7	0.59
*Juglans regia* L., Juglandaceae, XZH-201710298	Hetao	Sanmaizai	T	BA and RO	Decoction	Parasites; itching	SE and F	Seeds eaten fresh; flowers stir fried	4.9	0.91
*Clerodendrum bungei* Steud., Lamiaceae, XZH-20171069	Choumudan	Anikebazai	S	RO	Stewed with chicken	Rheumatism	RO	Stewed with chicken	2.2	0.41
*Elsholtzia bodinieri* Vaniot, Lamiaceae, XZH-201710132	Fengweicha	Sininongyuzai	H	WP	Soaked in boiling water	Iaryngopharyngitis	L and ST	Soaked in boiling water	2.7	0.51
*Elsholtzia ciliata* (Thunb.) Hyl., Lamiaceae, XZH-201710400	Xiangru	Yetongbingnizai	H	WP	Decoction	Headache; fever	L	Stewed with eggs; stir fried	2.3	0.48
*Elsholtzia flava* Benth*.*, Lamiaceae, XZH-201710231	Suzi	Ciwoguojiezai	H	FR	Decoction	Coughs; bronchitis	SE	Flavoring	2.6	0.48
*Elsholtzia rugulosa* Hemsl., Lamiaceae, XZH-201710358	Yebahao	Afeizai	H	WP	Decoction	Laryngopharyngitis	L and ST	Made tea	1.7	0.43
*Mentha canadensis* L., Lamiaceae, XZH-201710149	Bohe	Cihemiweizai	H	WP	Decoction	Pneumonia; relieving asthma	L and ST	Cold dish or boiled	4.2	0.88
*Holboellia latifolia* Wall., Lardizabalaceae, XZH-201710507	Maoshiguo	Mainaimezai	V	RO	Decoction	Cystitis	FR	Ripe fruits eaten fresh	2.3	0.49
*Litsea pungens* Hemsl., Lauraceae, XZH-201710448	Mujiangzi	Zuomeshengshengzai	T	RO	Decoction	Tonifying the spleen; clearing damp and treat gastritis	FR	Flavoring	4.9	0.90
*Parochetus communis* D. Don, Leguminosae, XZH-201710241	Jinquehua	Yetongshanmezai	H	F	Stir fried with eggs	Rheumatism; gastric disorders	F	Stir fried	2.6	0.54
*Pisum sativum* L., Leguminosae, XZH-201710551	Qingdou	Anuwadu	H	SE	Decoction	Gastralgia	SE	Boiled, eaten fresh or stewed	3.2	0.58
*Pueraria montana* (Lour.) Merr., Leguminosae, XZH-201710317	Ge	Zigaizai	V	RO	Decoction	Reducing phlegm; relieving alcoholism	RO	Eaten fresh	3.1	0.56
*Sophora davidii* (Franch.) Pavol., Leguminosae, XZH-201710126	Kucihua	Luocizai	S	F and RO	Decoction	Dysmenorrhea	F	Stir fried or boiled	2.1	0.42
*Styphnolobium japonicum* (L.) Schott, Leguminosae, XZH-201710394	Huaihua	Milongwujiezai	T	FR	Decoction	Clearing liver and improving vision	F	Stir fried or boiled	2.4	0.51
*Vicia faba* L., Leguminosae, XZH-201710193	Candou	Anuazai	H	SE	Decoction	Tonifying the spleen; detumescence	SE	Stir fried or boiled	3.8	0.71
*Lilium brownii* F.E.Br. ex Miellez, Liliaceae, XZH-201710341	Baihe	Amezai	H	FR and BU	Decoction	Tonifying the lung; reducing phlegm	BU	Stir fried or boiled	2.8	0.55
*Linum usitatissimum* L., Linaceae, XZH-20171091	Yama	Zhongzizai	H	SE	Made oil or powder	Hypertension; skin diseases	SE	Made oil or powder	2.3	0.45
*Punica granatum* L., Lythraceae, XZH-201710411	Shiliu	Sanbuzai	T	RO	Decoction	Parasites; dysentery	F and FR	Flowers stir fried or boiled, ripe fruits eaten fresh	2.6	0.52
*Toona sinensis* (Juss.) M. Roem., Meliaceae, XZH-201710547	Xiangchun	Longbotong	T	BA and RO	Decoction	Gynecopathy; dysentery	TS	Stir fried or cold dish	2.4	0.52
*Ficus tikoua* Bureau, Moraceae, XZH-201710116	Dibanteng	Cisanpianlianzai	V	RO	Decoction	Gonorrhea	FR	Ripe fruits eaten fresh	2.0	0.41
*Morus alba* L., Moraceae, XZH-201710184	Sangshu	Aheizai	T	RO	Decoction	Coughs; reducing phlegm	FR	Ripe fruits eaten fresh	2.6	0.53
*Musa basjoo* Siebold & Zucc. ex linuma, Musaceae, XZH-201710442	Bajiao	Gongguabuzai	T	RO	Decoction	Tonifying the spleen; diuresis	ST and F	Stir fried or boiled	3.5	0.65
*Myrica rubra* (Lour.) Siebold & Zucc., Myricaceae, XZH-201710129	Yangmei	Sangusongzai	T	RO	Decoction	Enteritis	FR	Ripe fruits eaten fresh	2.7	0.56
*Syzygium aromaticum* (L.) Merr. & L. M. Perry, Myrtaceae, XZH-201710222	Dingxianghua	Lazigumezai	T	RO	Stewed with pig trotter	Detumescence; analgesic	RO	Stewed with pig trotter	2.6	0.48
*Osmanthus fragrans* Lour., Oleaceae, XZH-201710402	Guihua	Jiweilongzai	T	F	Decoction	Moistening the lung; fever	F	Soaked or stir fried	2.8	0.58
*Nervilia fordii* (Hance) Schltr., Orchidaceae, XZH-201710544	Qingtiankui	Weinimesongzai	H	WP	Stir-fried with eggs	Hepatitis; detumescence	T	Stir fried or boiled	2.3	0.43
*Brandisia hancei* Hook. f., Paulowniaceae, XZH-201710274	Mitonghua	Dongmilongzai	S	RO and L	Decoction	Osteomyelitis	F	Stir fried or boiled	2.1	0.38
*Sesamum indicum* L., Pedaliaceae, XZH-201710111	Zhima	Guogeimiweizai	H	FR	Decoction	Deobstruent	SE	Eaten fresh or flavoring	2.9	0.55
*Keteleeria evelyniana* Mast., Pinaceae, XZH-201710275	Shasong	Geizai	T	BA and RO	Decoction	Fractures; detumescence	TS	Stir fried or boiled	2.2	0.46
*Pinus armandii* Franch., Pinaceae, XZH-201710432	Kongsong	Shumezai	T	BA, TS, SE, P, and RO	Decoction	Rheumatism; injury; coughs; deobstruent; gastritis	SE	Eaten fresh	2.1	0.42
*Pinus yunnanensis* Franch., Pinaceae, XZH-201710333	Yunnansong	Tumumezai	T	P, L, and BA	Decoction	Hyperglyceridemia	P	Made cakes	2.4	0.47
*Piper betle* L., Piperaceae, XZH-201710208	Luzi	Heicuonianmezai	V	ST, FR, and L	Decoction	Gastritis; coughs; eczema; oxytocin; anthelmintic	FR	Fruit chew with lime powder	2.3	0.48
*Phyllanthus emblica* L., Phyllanthaceae, XZH-201710522	Yuganzi	Elengmezai	T	FR, RO, and L	Decoction	Pharyngolaryngitis; moistening the lung; improve digestion; embolism; flatulence and coughs	FR, BA	Ripe fruits eaten fresh	4.9	0.93
*Plantago asiatica* L., Plantaginaceae, XZH-201710201	Cheqiancao	Geniwu	H	WP	Decoction	Cystitis	L	Stir fried or boiled	2.6	0.46
*Lophatherum gracile* Brongn. Poaceae, XZH-201710453	Zhuye	Medongzai	H	L	Decoction	Coughs; relieving asthma	TS	Stir fried or boiled	2.3	0.45
*Zea mays* L., Poaceae, XZH-201710511	Yumi	Huomezai	H	CS	Decoction	Removing urinary calculus	FR	Stir fried or boiled	4.9	0.93
*Fagopyrum tataricum* (L.) Gaertn., Polygonaceae, XZH-20171028	Kuqiaomai	Guokangmezai	H	WP	Decoction	Cholecystitis	L, TS and FR	Stir fried or boiled	3.1	0.60
*Ceratopteris thalictroides* (L.) Brongn., Pteridaceae, XZH-201710218	Shuijuecai	Riabiwujiezai	H	WP	Decoction	Diuresis	TL	Stir fried or boiled	2.9	0.54
*Ramalina fastigiata* (Pers.) Ach., Ramalinaceae, XZH-201710477	Shuhuacai	Sinengbazai	L	WP	Decoction	Tonifying the spleen; diuresis	WP	Stir fried or boiled	2.2	0.46
*Aconitum hemsleyanum* E. Pritz., Ranunculaceae, XZH-20171047	Caowu	Ciduzai	H	RO	Stewed with chicken	Rheumatism	RO	Stewed with chicken	0.6	0.05
*Hovenia acerba* Lindl., Rhamnaceae, XZH-201710250	Guaizao	Chenglenggalazai	T	FR	Decoction	Protective liver	FR	Ripe fruits eaten fresh	2.1	0.46
*Chaenomeles sinensis* (Dum. Cours.) Koehne, Rosaceae, XZH-201710174	Mugua	Sanbuzai	T	FR	Stewed with chicken	Numb limbs; rheumatism	FR	Boiled	2.9	0.60
*Crataegus cuneata* Siebold & Zucc., Rosaceae, XZH-201710324	Yeshanzha	Sanwozai	S	FR	Decoction	Hypertension; help digestion	FR	Ripe fruits eaten fresh	2.0	0.41
*Docynia delavayi* (Franch.) C. K. Schneid., Rosaceae, XZH-20171005	Duoyiguo	Sanbuazeizai	T	RO	Decoction	Rheumatism	FR	Ripe fruits eaten fresh	2.7	0.53
*Duchesnea indica* (Jacks.) Focke, Rosaceae, XZH-201710109	Shemei	Hamezai	H	WP	Decoction	Relieving asthma	FR	Ripe fruits eaten fresh	2.4	0.47
*Eriobotrya japonica* (Thunb.) Lindl., Rosaceae, XZH-201710428	Pipa	Chichishandongzai	T	L	Decoction	Coughs; reducing sputum	FR	Ripe fruits eaten fresh	2.8	0.67
*Fragaria × ananassa* (Duchesne ex Weston) Duchesne ex Rozier*,* Rosaceae, XZH-201710481	Hongshecao	Abosanzuinizai	H	WP	Decoction	Detoxifying snake venom; hemorrhoids	FR	Ripe fruits eaten fresh	2.4	0.47
*Fragaria nilgerrensis* Schltdl. ex J. Gay, Rosaceae, XZH-201710284	Baishecao	Abosangantong	H	WP	Decoction	Hepatitis	FR	Ripe fruits eaten fresh	2.3	0.48
*Fragaria vesca* L., Rosaceae, XZH-201710379	Fanbaiyecao	Laigusanzuitong	H	WP	Decoction	Detoxifying snake venom	FR	Ripe fruits eaten fresh	2.4	0.45
*Prinsepia utilis* Royle, Rosaceae, XZH-201710438	Qingciguo	Babazai	S	RO and L	Leaves Stir-fried with eggs	Cholecystitis; protect gastric mucosa; dry skin	TL and SE	Boiled or pickled; seed oil	2.7	0.54
*Prunus armeniaca* L., Rosaceae, XZH-201710255	Xingshu	Sanzuinizai	T	SE	Decoction	Coughs; reducing sputum	FR	Eaten fresh	2.7	0.51
*Prunus davidiana* (CarriŠre) Franch., Rosaceae, XZH-201710299	Yetao	Laigusanwuzai	T	SE	Decoction	Removing urinary calculus; analgesic	SE	Eaten fresh	2.2	0.47
*Prunus mume* (Siebold) Siebold & Zucc*.*, Rosaceae, XZH-201710270	Meizishu	Sangazimezai	T	RO	Decoction	Tonifying the spleen	FR	Ripe fruits eaten fresh	2.8	0.54
*Prunus pseudocerasus* Lindl., Rosaceae, XZH-201710519	Yingtao	Laiyumezai	T	RO	Decoction	Arthritis	FR	Ripe fruits eaten fresh	2.8	0.54
*Pyrus calleryana* Decne., Rosaceae, XZH-201710295	Douli	Sanlimesong	T	FR	Decoction	Acne	FR and F	Ripe fruits eaten fresh; flowers stir fried or boiled	2.4	0.43
*Pyrus pyrifolia* (Burm. f.) Nakai, Rosaceae, XZH-201710436	Yemianli	Sanlimiansong	T	FR and RO	Decoction	Tonifying the lung	FR and F	Ripe fruits eaten fresh or boiled; flowers stir-fried or boiled	2.3	0.46
*Rubus coreanus* Miq., Rosaceae, XZH-20171066	Maoyechatianpao	Sanhameazizai	S	RO	Decoction	Rheumatic arthritis	FR	Ripe fruits eaten fresh	2.2	0.42
*Rubus ellipticus* Sm., Rosaceae, XZH-201710101	Huangsuomei	Sanhamezai	S	RO	Decoction	Strokes; rheumatism	FR	Ripe fruits eaten fresh	2.0	0.40
*Rubus niveus* Thunb., Rosaceae, XZH-201710492	Heisuomei	Zinisan	S	RO	Decoction	Tonifying the liver; improving the vision; Sialaporia; rheumatism	FR	Ripe fruits eaten fresh	2.1	0.42
*Citrus medica* L., Rutaceae, XZH-201710138	Xiangyuan	Paoguozai	S	FR	Decoction	Enteritidis; stomachaches	FR	Ripe fruits eaten fresh	2.6	0.48
*Zanthoxylum armatum* DC., Rutaceae, XZH-201710425	Yehuajiao	Laiguzuozai	T	RO, BA, and FR	Decoction	Numb limbs; rheumatism	FR	Flavoring	2.3	0.43
*Zanthoxylum bungeanum* Maxim., Rutaceae, XZH-20171055	Huajiao	Zuozailongzai	T	RO and FR	Decoction	Skin itch	FR	Flavoring	3.3	0.59
*Houttuynia cordata* Thunb., Saururaceae, XZH-201710268	Yuxingcao	Awobinizai	H	WP	Decoction	Coughs; moistening the lung	WP	Cold dish or boiled	4.6	0.90
*Smilax mairei* H. Lév., Smilacaceae, XZH-20171033	Wucibaqia	Megulianzhongzai	S	RO	Decoction	Chronic colitis; irregular menses	TS	Stir fried or boiled	2.0	0.36
*Smilax riparia* A. DC., Smilacaceae, XZH-201710387	Maweicai	Memazhuzai	V	WP	Decoction	Fever; coughs	WP	Stir fried or boiled	2.1	0.33
*Capsicum annuum* L., Solanaceae, XZH-201710504	Lazi	Paizimezai	H	FR	Decoction	Diaphoretic	FR	Stir fried, boiled or pickled	4.6	0.90
*Physalis alkekengi* var. *franchetii* (Mast.) Makino, Solanaceae, XZH-201710375	Denglongguo	Amaishanbuzai	H	RO	Decoction	Pharyngolaryngitis; coughs	FR	Ripe fruits eaten fresh	2.8	0.50
*Pionandra betacea* (Cav.) Miers, Solanaceae, XZH-201710029	Shufanqie	Siazizai	T	FR	Decoction	Tonifying the spleen; stomach -aches	FR	Ripe fruits eaten fresh	2.4	0.47
*Camellia reticulata* Lindl., Theaceae, XZH-201710290	Shancha	Laiguzuomizai	S	FR	Decoction	Deobstruent	SE	Made oil	2.4	0.46
*Camellia sinensis* (L.) Kuntze, Theaceae, XZH-201710496	Cha	Longri	S	L	Soaked in boiling water	Hyperglyceridaemia	TL	Soaked in boiling water	4.3	0.82
*Debregeasia orientalis* C. J. Chen, Urticaceae, XZH-20171075	Shuima	Yuzimezai	S	RO and ST	Decoction	Dysentery	FR and F	Stir fried or boiled	1.8	0.32
*Urtica atrichocaulis* (Hand.-Mazz.) C.J. Chen, Urticaceae, XZH-201710288	Xiaoqianma	Dengbuazizai	H	WP	Stir fried with eggs	Prostatitis	TS	Stir fried or boiled	1.7	0.28
*Ampelopsis delavayana* Planch. ex Franch., Vitaceae, XZH-201710420	Yeputao	Runimesanzai	V	RO	Decoction	Fractures; analgesic	FR	Ripe fruits eaten fresh	2.1	0.40
*Amomum tsao-ko* Crevost & Lemarié, Zingiberaceae, XZH-20171087	Caoguo	Sibiyumezai	H	FR	Decoction	Renal colic	FR	Flavoring	3.3	0.65
*Zingiber officinale* Roscoe, Zingiberaceae, XZH-201710371	Jiang	Chibozai	H	FR	Decoction	Headache; diaphoretic	RH and ST	Flavoring	4.5	0.87

Life form: *H* Herb, *S* Shrub, *T* Tree, *V* Vine, *F* Fungus, *L* Lichen. Medicinal parts and edible parts: *WP* Whole plant, *RH* Rhizome, *T* Tuber, *ST* Stem, *RO* Root, *BA* Bark, *L* Leaf, *FB* Fruit body, *F* Flower, *SE* Seed, *CS* Corn silk, *BU* Bulb, *P* Pollen, *TS* Tender shoot, *TL* Tender leaf, *TP* Tender plant, *FR* Fruit

The order of plant species in this table is followed by the APG IV system, gymnosperms classification system (1978), and Qinrenchang fern plant classification system (1978). Specimens were deposited in the Herbarium of the Key Laboratory of Chemistry in Ethnic Medicinal Resources, State Ethnic Affairs Commission & Ministry of Education, P. R. China, School of Ethic Medicine, Yunnan Minzu University, Kunming, China

**Table 3:** Taxonomic distribution of medicinal dietary plants used by Yi in Mile City, Yunnan, China

Plant group	Number of species	Number of genera	Number of families
Angiosperms	117	96	57
Gymnosperms	3	2	1
Pteridophytes	2	2	2
Lichen	1	1	1
Fungus	1	1	1
Total	124	102	62

**Fig. 3:**
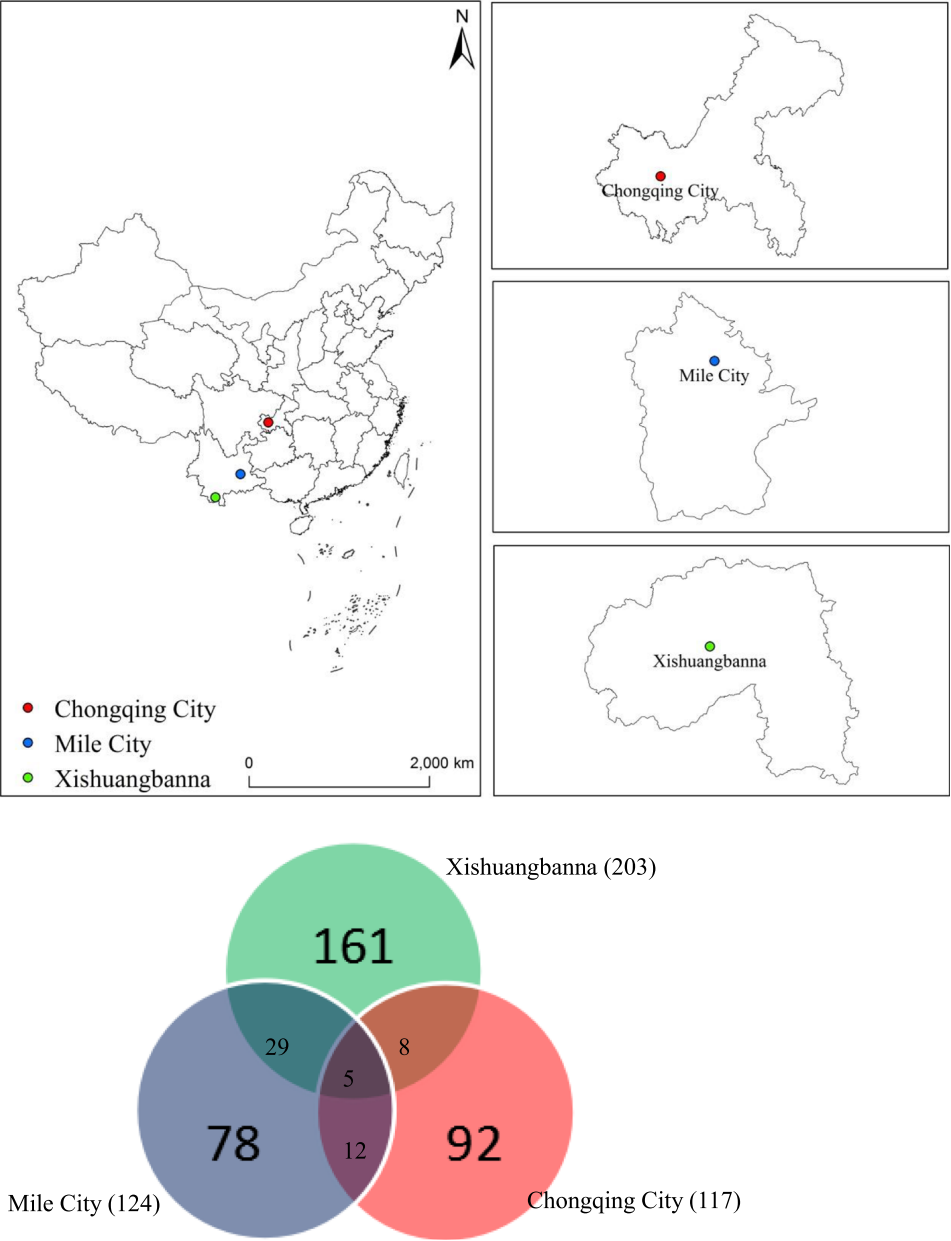
Venn diagram comparing plant species used for food medicine in this research to those found in similar studies conducted in Xishuangbanna and Chongqing

In the Mile area, 124 plant species were used as medicinal dietary, whereas the results of previous studies showed that the number of plant species was relatively low in Jinghong, Mengla, and Menghai of Xishuangbanna [22, 35, 36] and the number was relatively high in Jinfoshan of Chongqing [3]. The overlap between these areas was illustrated using a Venn diagram (Fig. 3).

### The UV and FUI values of medicinal dietary plants in Mile City

Quantitative analyses were performed to determine the local importance of each medicinal dietary plant. The UV and FUI for each species were calculated (Table 1). The 20 medicinal dietary plant species with the highest UV were listed along with their FUI in Table 4. *Amorphophallus konjac*, *Cucurbita moschata*, *Phyllanthus emblica*, and *Zea mays* had the highest UV and FUI (Table 4). Their UV and FUI were 0.93 and 4.90, respectively. This result demonstrated that these plants had major importance for local people.

**Table 4:** Top 20 medicinal dietary plants with highest use value in Mile City

Scientific name	Medicinal uses	FUI	UV
*Amorphophallus konjac*	Coughs	4.90	0.93
*Cucurbita moschata*	Acaricide	4.90	0.93
*Phyllanthus emblica*	Pharyngolaryngitis; coughs	4.90	0.93
*Zea mays*	Removing kidney stones	4.90	0.93
*Juglans regia*	Anthelmintic; relieving itching	4.90	0.91
*Litsea pungens*	Gastritis	4.90	0.90
*Allium sativum*	Rhinitis; anthelmintic	4.70	0.90
*Houttuynia cordata*	Coughs; moistening the lung	4.60	0.90
*Capsicum annuum*	Diaphoretic	4.60	0.90
*Auricularia auricula*	Deafness; hypertension	4.20	0.89
*Taraxacum mongolicum*	Analgesic; diuretic	4.50	0.89
*Sechium edule*	Gastritis; colitis	4.50	0.88
*Mentha canadensis*	Pneumonia; relieving asthma	4.20	0.88
*Zingiber officinale*	Headache; Diaphoretic	4.50	0.87
*Oenanthe javanica*	Hypertension	4.20	0.83
*Camellia sinensis*	Hyperglyceridemia	4.30	0.82
*Vicia faba*	Tonifying the spleen; edemas	3.80	0.71
*Eriobotrya japonica*	Coughs; reducing sputum	2.80	0.67
*Musa basjoo*	Tonifying the spleen; diuretic	3.50	0.65
*Amomum tsao*-*ko*	Renal colic	3.30	0.65

Several other plants in our study area were found to be popular as food medicines based on their high UV and average FUI, including the following: *Juglans regia*, *Litsea pungens*, *Allium sativum*, *Houttuynia cordata*, *Capsicum annuum*, *Auricularia auricula*, *Taraxacum mongolicum*, *Sechium edule*, *Mentha canadensis*, *Zingiber officinale*, *Oenanthe javanica*, *Camellia sinensis*, *Vicia faba*, *Eriobotrya japonica*, *Musa basjoo*, and *Amomum tsao*-*ko.*

### Plant part used

**Fig. 4:**
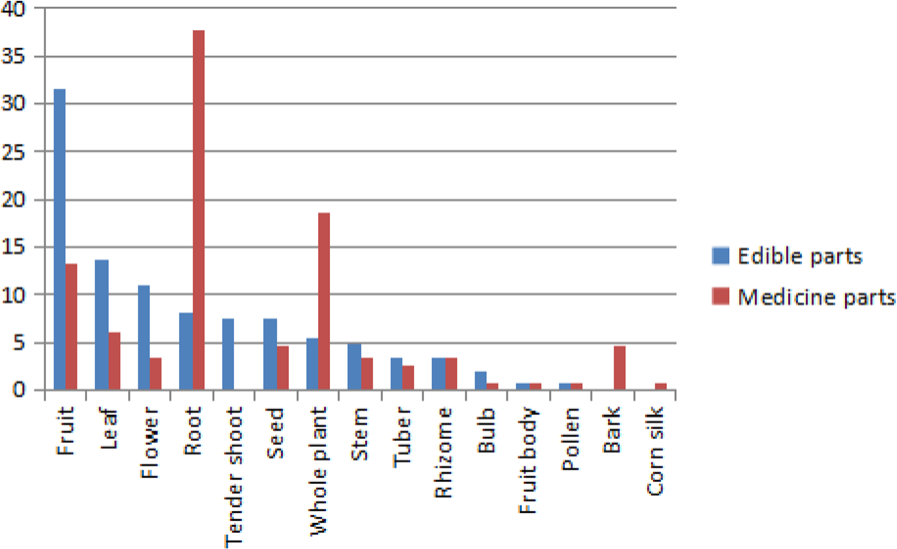
Frequency of use of the edible and medicinal parts of medicinal dietary plants by the Yi in Mile City, Yunnan, China

The most frequently used edible parts were the fruits, leaves, flowers, roots, tender shoots, seeds, and the whole plant (Fig. 4). There were 46 types of edible fruits among the 124 medicinal dietary plants documented here. This might be the reason why the fruits were easily collected, and ripe fruits usually had a good taste, which was readily accepted by people as food. In addition, the edible parts of many plants were flowers. For example, the flowers of *Marsdenia tenacissima* and *Rhododendron mucronatum* were often prepared by boiling and frying, which was a typical feature of the Yi dietary culture. In addition, certain parts of other species, such as the bark of *Phyllanthus emblica*, the seed oil of *Prinsepia utilis*, and the pollen of *Pinus yunnanensis*, were recognized as edible items.

Certain portions of those plants, namely the roots, fruits, tender shoots, and even the whole plants, had been used for thousands of years and reportedly play therapeutic roles in treating many diseases (Fig. 4). For instance, the roots from 57 (approximately 46%) of the 124 documented plants were used as restorative treatments. The book *Essentials of Yi Medicine* indicated that Yi doctors were particularly good at using plant roots for medical treatment. Moreover, 82.48% of the roots used in Yi medicine were from herbs and were still not recorded in the literature on traditional Chinese or Tibetan medicine. The skill of Yi doctors in using roots might have developed from ancient Yi medical theories, such as the concepts of Qingzhuo, Bafeng of Yi, and the five elements of Yi [9].

### Preparation methods and cooking methods

The primary medicinal preparation method for the plants recorded in this study was decoction. However, 28 species required specific preparation methods (Table 2). For example, the Yi people used the rhizomes of *Dobinea delavayi* stewed with chicken to treat dizziness and alleviate fatigue and edemas, and they used the rhizomes and roots of *Saruma henryi* stewed with chicken or pig trotter to tonify the spleen and treat stomachaches. This approach reflected the efficient combination of food and drugs in the Yi medical system, which made it possible to achieve the objectives of health care and diseases treatment and prevention via the daily diet. The Yi medicine book, *Theory and Aplication of Yi Medicine* by Yi medicine expert Zhengkun Wang, recorded that the Yi people used specific preparation methods to maintain their health [20]. For example, it was necessary to know which material should be stewed with the herbs, chicken, or pig’s feet and how long the course of treatment should be. Sufficient knowledge about the herbs or materials and the preparation methods were required to achieve health protection.

The primary cooking methods used for the plants documented in this survey were boiling, followed by stir frying, and eating fresh. Three species of the above plants, *Amorphophalms konjac*, *Pinus yunnanensis*, and *Elsholtzia rugulosa*, were prepared by using specific cooking methods before eating and converted into konjac tofu, cakes, and tea, respectively. *Elsholtzia flava* also required special preparation. The seeds of *E*. *flava* were eaten as a seasoning in this area, and thus they had to be smashed or pressed to release their flavor adequately.

### Diseases treated by Yi medicinal dietary plants

The medicinal dietary plants used by the Yi people were diverse and contributed to the treatment of a number of disorders, such as coughs, rheumatism, edemas, kidney deficiency, spleen deficiency, gastritis, and parasites (Table 5) [37]. These diseases were widespread among the ethnic groups living in the mountains. The Yi people were prone to rheumatism and respiratory diseases due to the humid air, and they were often injured at work. Urinary tract and digestive system diseases were also frequent among the Yi. Modern Yi medicine developed from years of experience with environmental hazards and disease.

**Table 5:** Frequency of ailments treated with medicinal dietary plants in Mile City

Disease	Species used	Percentage (%)
Coughs	21	16.8%
Rheumatism	11	8.8%
Edemas	10	8%
Tonifying the kidney	10	8%
Tonifying the spleen	10	8%
Gastritis	10	8%
Anthelmintic	8	6.4%
Diuretic	6	4.8%
Pharyngitis	5	4%
Hepatoprotective	5	4%
Hypertension	5	4%
Moistening the lung	5	4%
Fever	5	4%
Hepatitis	5	4%
Enteritis	5	4%
Analgesic	4	3.2%
Asthma	4	3.2%
Cystitis	4	3.2%
Pneumonia	3	2.4%
Emmenagogue	3	2.4%
Diarrhea	3	2.4%
Deobstruent	3	2.4%
Migraines	3	2.4%
Indigestion	2	1.6%
Lactation stimulant	2	1.6%
Deafness; tinnitus	2	1.6%
Acne	2	1.6%
Kidney stones	2	1.6%
Itching	2	1.6%
Snake bites	2	1.6%
Sialaporia	2	1.6%
Fractures	2	1.6%
Hyperglyceridemia	2	1.6%
Cholecystitis	2	1.6%
Diaphoretic	2	1.6%
Pustules	1	0.8%
Anemia	1	0.8%
Embolism	1	0.8%
Tranquillizer	1	0.8%
Epilepsy	1	0.8%
Restorative	1	0.8%
Jaundice	1	0.8%
Nephritis	1	0.8%
Vomiting	1	0.8%
Purifier	1	0.8%
Bronchitis	1	0.8%
Alcohol poisoning	1	0.8%
Rhinitis	1	0.8%
Gonorrhea	1	0.8%
Angina	1	0.8%
Dizziness	1	0.8%
Hemorrhoids	1	0.8%
Strokes	1	0.8%
Flatulence	1	0.8%
Prostatitis	1	0.8%
Osteomyelitis	1	0.8%
Oxytocin	1	0.8%

The categories of ailments follow the standard: the Economic Botany Data Collection Standard (EBDCS) (Cook, 1995) [37]

## Discussion

### Medicinal dietary plants are abundant in Mile City

The Yi people have lived in Mile City for a long time. During their long struggle for survival, they have developed unique dietary and medication habits, and a large amount of folk knowledge about the use of medicinal dietary plants has been accumulated. Mile City is in the Honghe Hani and Yi Autonomous Prefecture, and the traditional knowledge of plants among the Yi is relatively well preserved. The intangible cultural heritages such as “A'Xi (Ah-shi) Dance in the Moon,” “A'Xi sei ji,” and “A'Xi sacrifice fire” originated and were passed on here. The minority nationality dominated by the Axi people of the Yi branch accounts for 94% in the town of Xiyi, which is the hometown of the national song and dance “A'Xi (Ah-shi) Dance in the Moon” and the birthplace of “A'Xi sacrifice fire.” Xier town is the birthplace of “A'Xi sei ji” of The Yi branch. The town of Xisan is known as the “kingdom of plants” and is home to the ecological park of northern Mile City. Trees, shrubs, vegetation, ferns, and mosses can be seen everywhere. The forest coverage rate reaches 71% [25]. The special geographical area and relatively independent living environment of the Yi people make it rich in medicinal dietary plants. In addition, due to the entry of the Han people into Yunnan, the Central Plains culture has been introduced into Mile City. The Mile Yi interacts with other cultures more frequently and widely than ever before. Consequently, some Yi people have learned about valuable medicinal and edible species through interaction and trading herbs with the Han Chinese. Maize, pumpkin, and other traditional medicinal dietary plants from the Central Plains have entered the life of the Yi in Mile City. The Yi people living in many places in China have the habit of using medicinal dietary plants. For example, the Yi who live in Xishuangbanna (Lancang River Basin), in southern Yunnan Province, and Liangshan, in Sichuan, also have the custom of using medicinal dietary plants to strengthen their physical health and prevent disease [38]. These individuals collect many medicinal WEPs for meals and stew them with pork every year during the Dragon Boat Festival [39], a holiday that was adopted from the Han Chinese. Similarly, the Yi, Lahu, and Han people in the Simao area of Yunnan Province, as well as the Zhuang people of Guangxi, have related traditions of eating meals of medicinal roots during the Dragon Boat Festival [40, 41].

### Medicinal dietary plants worldwide

In 2002, the Ministry of Health of the People’s Republic China published a “Notice on further standardizing the management of health food raw materials.” In this notice, specific provisions were made for 87 species of medicinal dietary plants, the items that can be used as health foods, and the articles prohibited for use as health foods. Although this provision has a guiding role in the development and utilization of medicinal dietary plants and plants to boost health, it must still be supplemented. The Yi people’s traditional knowledge of the medicinal dietary plants of their area is the accumulation of generations of wisdom and experience. Although many of the medicinal dietary plants documented in this study are not included in the notice, they are efficacious in the prevention and treatment of diseases. Therefore, it is of great importance to investigate the medicinal dietary plants in minority areas, to understand relevant traditional knowledge, to study the nutritional value and efficacy of the plants, and to rationally develop these plant resources to better serve human health [42].

Currently, more than 80% of the global population relies on traditional medicine for primary health care [28]. The use of medicinal dietary plants is an important form of health care for minority communities in remote areas. The Yi people use plant properties in the areas of food, health care, and medicine. In addition to providing food and nutrition, medicinal dietary plants can regulate human body functions due to the secondary metabolites they contain [3, 22, 43]. In Indian systems of traditional medicine, dietary recommendations are an integral and important part of the therapy; it is considered an ally for strengthening drug efficacy [4, 44].

At present, people all over the world are turning to healthy foods that supply good nutrition and prevent disease, which has led to the development of the health-food industry. While many traditional medicinal dietary plants have health functions, not all of them are suitable for everyone. Some of these plants contain toxins, and dangerous side effects may occur if they are used inappropriately. Therefore, before eating these plants, people should understand their effects, potential side effects, and the category of individuals who can safely consume them. Only in this way can traditional medicines be used efficaciously and with minimal side effects [6].

### Comparison among Chongqing (Jinfoshan), Mile City, and Xishuangbanna (Jinghong, Menghai, and Mengla)

The plant species used as medicinal dietary were compared among Jinfoshan, Mile City, and Xishuangbanna using a Venn diagram. It is clear that the diversity of medicinal dietary plants in different ethnic minority areas in China is very rich. Although there were some crossovers, they likely reflected that different ethnic groups in different regions had unique characteristics in relation to their use of medicinal dietary plants. Each area had its own knowledge. Xishuangbanna is located in the biodiversity center of China, which is rich in plant species. It has been inhabited by Dai, Jinuo, Hani, Yao, and Lahu for a long time. These ethnic groups have accumulated diverse and unique experience in using plants. Therefore, medicinal dietary plant species of the Xishuangbanna area are the most abundant. Both Mile City and Xishuangbanna belong to Yunnan, and there are a relatively large number of exchanges of medicine food culture among different ethnic groups, leading to great overlap in medicinal plant use. Mile City is relatively far from Jinfoshan in Chongqing and is separated by mountains, so the plants used there are inclined to their own characteristics, with fewer crossovers. This observation shows that the idiosyncratic nature of traditional knowledge is closely related to medicinal dietary plants.

### Four special cases of medicinal dietary plants in Mile City

Our survey found that four plants were used differently. *Amorphophallus konjac* is a popular food in the daily life of the Yi people*.* It contains a pharmacologically active heteropolysaccharide, konjac glucomannan (KGM), which is extracted from konjac tubers. KGM has the characteristics of water absorption, gelatinization, adhesiveness, and low-heat edibility, so it is widely used in food processing, pharmaceuticals, and health care products [45]. This tuber is used to make konjac tofu, which can be stir fried with Chinese sauerkraut or chicken or used in a cold dish. KGM can also be used for detoxification and to treat edemas, phlegm, bronchitis, and coughs [46].

**Fig. 5:**
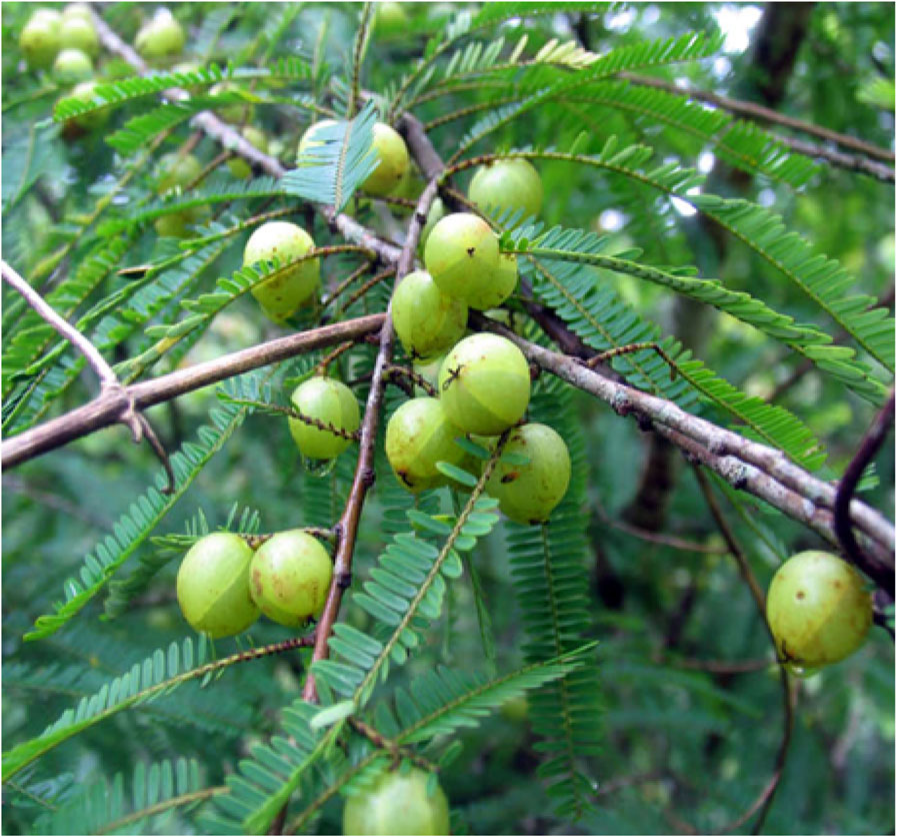
Fruits of *Phyllanthus emblica*

*Phyllanthus emblica* is widely distributed in the subtropical and tropical areas of China, India, Indonesia, and Malaysia. *Phyllanthus emblica* fruit is well accepted by consumers for its special taste. It has abundant amounts of vitamin C and superoxide dismutase, and it is used in many traditional medicinal systems such as Chinese herbal medicine, Tibetan medicine, and Yi preparations of medicinal dietary plants [47]. The flavor of the fruit is unique, with an initial sour taste and then a sweet taste. The Yi people like to eat the bark. They remove the bark from the fresh trunk and scrape the endothelium with a ceramic implement or knife to obtain ribbon-thin slices, which can be used in a cold dish or stir fried with meat [32]. *Phyllanthus emblica* fruit is also made into a drink (Fig. 5). In addition, it can moisten the lungs, improve digestion, and cure diseases such as embolism, flatulence, and coughs.

The fruit of *Litsea pungens* contains aromatic oil. The oil contents of its dried and fresh fruit are 2–6% and 3–4%, respectively. The primary ingredients of those two oils are citric aldehyde 60–90% and geraniol 5–19%, which can be used as edible essence [48]. It also has medicinal functions, such as tonifying the spleen, clearing the damp, and treating gastritis.

**Fig. 6:**
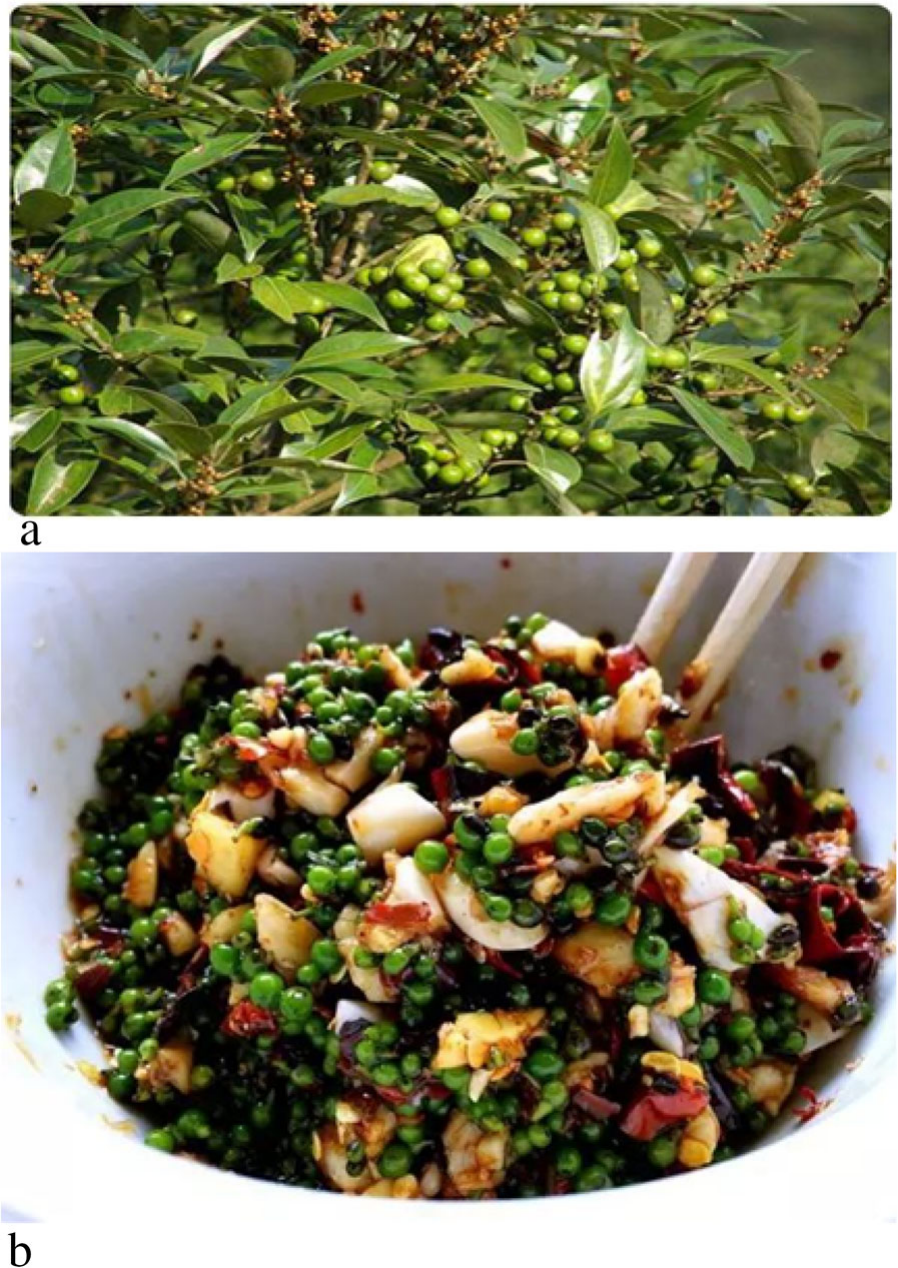
Fruits of *Litsea puens* (**a**) and dishes containing *Litsea pungens* (**b**)

The fruits are lightly patted into pieces and salted with salt and soy sauce and then allowed to marinate for a while. After that, the fruit is mixed with garlic paste and can be eaten. According to the above edible method, these fruits can be made into a delicious cold dish that preserves their original taste. The fruit is stir fried with fresh beef and is also a delicacy of the local Yi people (Fig. 6).

*Piper betle* is an indigenous climber of the Mile region. Its ethnomedicinal application has been well known for a long time. It is traditionally used to cure odontalgia, wind-cold coughs, bronchial asthma, rheumatism, stomachalgia, and edema of pregnancy, and it is an aphrodisiac and has anticancer properties [49]. In addition, decoctions from the *P*. *betle* fruits are used for its oxytocin by local Yi people, and its fruits, when mixed with a small amount of lime, are usually chewed by local people to protect the teeth.

### Special preparation and cooking methods

Generally, traditional preparation methods of medicinal plants are similar to those normally used in cooking; however, some poisonous plants need special preparation. *Aconitum hemsleyanum* is a highly toxic plant because it contains aconitine. The preparation process is strictly controlled to protect against toxic effects. The specific cooking process is as follows: the water must be boiled completely, and then fresh aconitum root and lard is placed in the water and constantly boiled for at least 24 h. Boiling water must be added to prevent the water from evaporating, ensure food safety, and preventing food poisoning. In addition, people should stay in a warm room for one night after eating the concoction. This processing method is different from previous report [50, 51].

More than 13 cooking methods were documented in this survey. During their long history, the local Yi people have learned how to use the edible and medicinal parts of wild plants in the most effective ways. Another plant that needs careful handling is *Pteridium aquilinum*. The tender leaves of this fern are a popular wild food in the area. However, improper handling may lead to poisoning. *P*. *aquilinum* contains ptaquiloside, which is harmful to humans and animals if eaten raw. Ptaquiloside has been listed as a class III carcinogen by the IARC (International Agency for Research on Cancer) [52, 53]. To avoid their harmful effects, the leaves are cooked in water for a long time with frequent water changes until they are very soft, and then stir fried.

### Comparison medicinal and dietary parts

Among the 124 plants documented in this study, 40 species had edible parts that were used for food medicine. In 82 species, however, the parts used for medicine and the parts used for food were different. For example, the tender shoot of *Aralia chinensis* can be eaten after frying or boiling, while a decoction of the bark and roots can tonify the liver and treat rheumatism. From *Zea mays*, the fruit is eaten, and the corn silk has medicinal properties. It was observed that through the long-term use of these plants, the Yi people had a very thorough understanding of the characteristics and effects of the various plant parts, and so they made the best use of these resources. The preference for wild-collected leafy vegetables and fruits over underground plant parts for food seems to be common among diverse ethnic groups in Mile City and might be due to the ease of collecting the above-ground parts; see Fig. 4 and Table 2. The most frequently used medicinal parts of these plants are roots, which may be due to their relatively high medicinal content.

Other ethnic groups might use some medicinal plants in different ways compared to those employed by the Yi. Zhang et al. recorded 55 species of medicinal dietary plants used by the Naxi people in the Lijiang area [2]. Among them, 11 species were recorded as being used in a different way. The medicinal parts, preparation methods, and efficacy of those 11 plants in Yi medicine were quite different from those recorded in Naxi medicine. For example, the Yi people preferred to decoct the roots of *Foeniculum vulgare* to treat traumatic injuries, abdominal distension, and stomachaches, while the Naxi people were more likely to use the tender stems of *F*. *vulgare* steamed together with eggs to alleviate fatigue and backache. Notably, although ethnic medicine was similar to traditional Chinese medicine in medical outcomes, the various systems used by the different ethnic groups with their specific methods and characteristics are attracting more and more attention from researchers.

### Pharmacological properties

Drugs derived from plants or their extracts have specific therapeutic properties. If antibiotics are replaced with suitable therapeutic agents, plants can play an important role in combating bacterial pathogens. In this section, we will analyze the pharmacological properties of the most frequently used medicinal plant species to check their therapeutic efficacy. This is important because antibiotic resistance is an emerging global concern and a research hotspot with respect to veterinary and human medicine.

The tuber of *Amorphophallus konjac* is used to remove toxicity and to treat edemas, gastritis, bronchitis, and persistent coughs [54]. The tuber of this herb contains glucomannan, starch, proteins, amino acids, and impurities. Glucomannan is an ideal dietary fiber and contains 7 essential amino acids. Four types of serotonin compounds, which have the ability to inhibit inflammatory cytokines, were isolated from the flying powder. Notably, the tuber of *Colocasia esculenta* has 31 alkanes, pea sterol, palmitic acid, carotenoids, and other components. It contains 19 mineral elements, 17 free amino acids, 7 essential amino acids, water-soluble polysaccharides, aromatic alcohols, and oxalates. The fruit of *Phyllanthus emblica* has been used as a medicine in China for at least 2000 years. The roots and leaves of the tree can be used for medicinal purposes, such as moistening the lungs, improving digestion, and curing diseases such as embolism, flatulence, and coughs. Pharmacological studies have shown that it has anti-microbial and anti-oxidant effects and lowers blood lipids and blood glucose levels. The fruits are rich in vitamin C and carotene, and the seeds are rich in fatty acids, phospholipids, and essential oils. The fruit, bark, and leaves contain tannins and can be used for treating diarrhea. Six polyphenolic compounds are obtained from *P*. *emblica* fruit juice.

The flowers of *Camellia sinensis* can be used as a medicine and have an astringent effect, and the leaves can be used as a substitute for tea. The oil from the seeds contains 5 types of fatty acids and has an unsaturated fatty acid content of more than 80%. It also contains 8 types of mineral elements, including iron, sodium, and magnesium, and it can be used as a tonic. In addition, the plant contains flavonoids, polyphenols, and tea glycosides. Another plant with multiple medicinal uses is *Pinus armandii.* Local people use the shoots of *P*. *armandii* to treat rheumatism and injury. The seed kernels are used to treat the coughs caused by lung heat (a syndrome in Chinese medicine) and deobstruent, while the pollen is used to treat gastritis. *P*. *armandii* seeds have an oil content of 56.5%, which includes linoleic acid, oleic acid, palmitic acid, and arachidonic acid. In one study, the unsaturated fatty acids of *P*. *armandii* seeds had a significant inhibitory effect on hyperlipidemia and arteriosclerosis in mice [55].

### Commercial potential

Many of the plants documented in this study have potential for development because of their low toxicity and significant medicinal efficacy. For example, the roots of *Oenanthe javanica* can be used to treat hypertension. The roots of *Cirsium japonicum* and *Cirsium arvense* are highly effective at tonifying the kidney. The fruits of *Cornus kousa* subsp. *chinensis* belong to an active class of acaricide that is poisonous to mites and ticks. The oil and powder from the seeds of *Linum usitatissimum* can treat various skin diseases. The roots of *Myrica rubra* are a good source of medicine to cure enteritis. The roots of *Prinsepia utilis* showed good anti-inflammatory activity in cholecystitis. Its seeds are rich in oil, edible, and contain various nutrients. The oil is a high-grade natural edible oil with uses in food medicine. It can protect the gastric mucosa and is a good treatment for dry skin. Thus, *P*. *utilis* has great potential for development as a raw material for natural skin care and health care products [56].

Apart from their use in food medicine, many of the species in this study were put to multiple other uses. For example, many of them are used as ornamental plants or made into teas. Wild food plant species are abundant and diverse in Mile City, they provide food for the local people, and they are also a source of income. People are paying more attention to food safety and the preservation of health. Because of their excellent beneficial effects in disease prevention and treatment, medicinal dietary plants should be developed as health products or drugs [57].

### Conservation issues

With the rapid development of the economy and an accelerating loss of biological and cultural diversity, a large amount of the traditional knowledge of minority nationalities is in danger of disappearing. Therefore, the documentation and evaluation of traditional knowledge related to plant diversity and the use and effects of medicinal dietary plants are crucial [58, 59]. For example, plants such as *Dobinea delavayi*, *Saruma henryi*, *Adenophora stricta*, *Aconitum hemsleyanum*, and *Codonopsis cordifolioidea* are scarce wild resources. Many precious plant resources that have the potential for future sustainable development are vanishing before they have even been discovered. The development of plant resources is necessary to maintain biological diversity and for the potential development of drugs and health care products. In addition, the loss of traditional knowledge has been recognized as a development that has important negative effects on biological diversity conservation [60]. A reduction in plant diversity also leads to the extinction of the associated indigenous knowledge. Over-harvesting may have serious consequences for both plant survival and the environment. The conservation and sustainable use of species with multiple uses must be taken into consideration. Admittedly, the overexploitation of these resources has led to some degree of protection through cultivation. The artificial cultivation of *Eleutherococcus senticosus* and *Prinsepia utilis* is already performed and provides a model for the sustainable use of plant resources.

## Conclusion

This is the first ethnobotanical research study on the medicinal dietary plants used by the Yi ethnic group in Mile City, Yunnan, China. Yunnan is rich in biodiversity thanks to its favorable geographical conditions, which endow the Yi with medicinal dietary plants with distinct characteristics.

Quantitative analyses were performed to determine the local importance of each medicinal dietary plant. The UV and FUI for of each species were calculated. The 20 medicinal dietary plant species with the highest UV are listed along with their average FUI in Table 4. *Amorphophallus konjac*, *Cucurbita moschata*, *Phyllanthus emblica*, and *Zea mays* had the highest UV and average FUI.

The primary medicinal preparation method for the plants recorded in this study was decoction. However, the 28 species required specific preparation methods. The primary cooking method for the plants documented in this survey was boiling, followed by stir frying and eating fresh. The most frequently used edible parts were fruits, leaves, flowers, roots, tender shoots, seeds, and the whole plant. There were 46 types of edible fruits among the 124 medicinal dietary plants documented here. Regarding the medicinal parts of these plants, some roots, whole plants, and fruits have been used for thousands of years and are reported to possess certain medical effects. The medicinal dietary plants used by the Yi people are diverse and contribute to the treatment of a number of disorders, such as coughs, rheumatism, edemas, kidney deficiency, spleen deficiency, gastritis, and parasites.

Ethnobotanical surveys of medicinal dietary plants provide a theoretical reference for the conservation and sustainable use of the plant resources and can contribute to the protection of the Yi food culture and traditional medicine in Mile City. In addition, this information provides a sound basis for the development and use of Yi ethnic medicine and health products.

There are some limitations in our study. For example, relatively few studies have been performed on the pharmacology, toxicology, and adverse effects of edible medicinal plants used by the Yi people. In future investigations, we will perform more researches to obtain more comprehensive information.

## Data Availability

All data generated or analyzed during this study are included in this published article (and its supplementary information files).
